# A protocol for ploidy determination in ethanol-fixed Atlantic salmon (*Salmo salar*) fry using propidium iodide and internal standardization: Compatible with field conditions at the sample preservation stage

**DOI:** 10.1016/j.mex.2026.104143

**Published:** 2026-09-04

**Authors:** Sebastián Ávila, Maira Toro, Wellison Amorim Pereira, Christian Ellwanger-Reyes, Iván Valdebenito Isler, Elías Figueroa-Villalobos

**Affiliations:** a Doctorado en Ciencias Agropecuarias, Facultad de Recursos Naturales, Universidad Católica de Temuco, Temuco, Chile; b Facultad de Ciencias Agropecuarias y Medio Ambiente, Universidad de La Frontera, Temuco, Chile; c Laboratory of Microbial Biomolecules, School of Pharmaceutical Sciences, University of São Paulo, São Paulo, Brazil; d Departamento de Ciencias Agropecuarias y Acuícolas, Facultad de Recursos Naturales, Universidad Católica de Temuco, Temuco, Chile; e Núcleo de Investigación en Producción Alimentaria, Facultad de Recursos Naturales, Universidad Católica de Temuco, Temuco, Chile

**Keywords:** Flow cytometry, Ploidy determination, Salmo salar, Propidium iodide, Ethanol fixation

## Abstract

Ploidy verification in salmonid production is often constrained by the need to analyze fresh samples. Here, we present an optimized protocol for early-stage ploidy determination in Atlantic salmon (Salmo salar) fry using 70 % ethanol fixation at −20 °C and flow cytometry. A staining solution containing Propidium Iodide (PI) and RNase A was employed to ensure stoichiometric DNA quantification, combined with internal biological standardization to minimize instrumental variation. The protocol achieved a 92.7 % analytical measurability rate and a mean coefficient of variation (CV) of 3.10 %, comparable to values reported for fresh blood samples. The normalized fluorescence ratio discriminated all valid samples, yielding values of 1.00 ± 0.05 for diploids (2n) and 1.38 ± 0.04 for triploids (3n) produced by thermal shock applied at 15 and 30 min post-fertilization. Cross-validation by nuclear morphometry confirmed a significant increase in the major nuclear diameter of putative triploids (7.69 ± 1.04 µm) relative to diploids (6.63 ± 0.83 µm). However, an area under the curve (AUC) of 0.76 indicated lower diagnostic performance than flow cytometry. This protocol provides a practical method for ploidy assessment in Atlantic salmon fry, compatible with field conditions at the sample preservation stage

## Highlights

• Enables reliable ploidy determination in Atlantic salmon fry using ethanol-fixed blood samples.
• Uses internal biological standardization to improve fluorescence normalization and reduce analytical variability.
• Enables field preservation of aquaculture samples for later lab analysis.

## Graphical abstract

Graphical abstract was submitted as a separate file.

**Figure:**
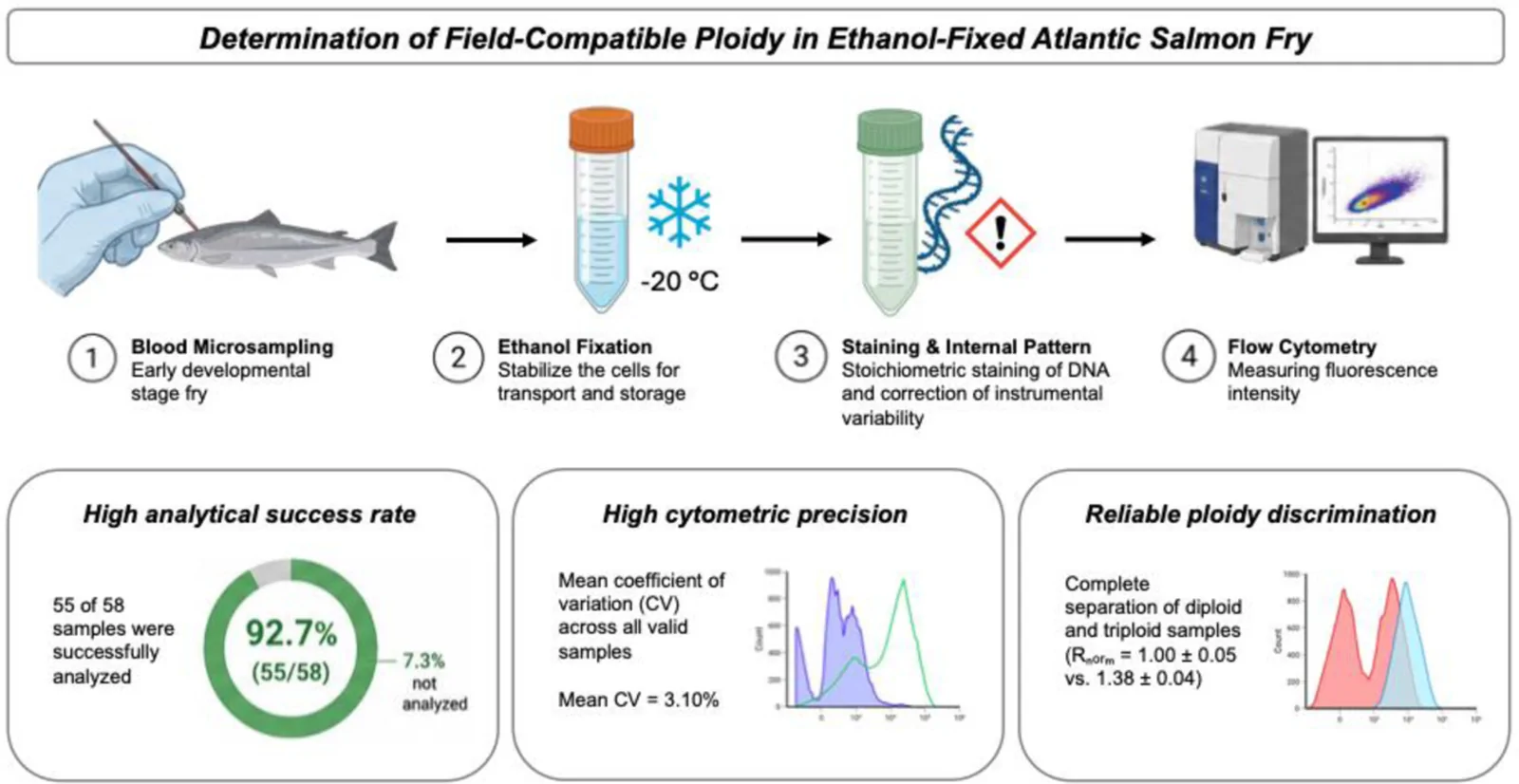

**Specifications table**

**Table:** 

**Subject area**	Agricultural and Biological Sciences
**More specific subject area**	Aquaculture, Fish Genetics, Flow Cytometry
**Name of your method**	Flow cytometric ploidy determination in ethanol-fixed Atlantic salmon (Salmo salar) fry using propidium iodide staining and internal biological standardization
**Name and reference of original method**	Flow cytometric DNA content analysis using propidium iodide staining and internal standards, adapted from Darzynkiewicz and Huang (2004), Vindeløv et al. (1983), and Hubálek and Flajšhans (2021)
**Resource availability**	The protocol uses standard laboratory reagents (70% ethanol, RNase A, propidium iodide) and a flow cytometer equipped with a red laser and FL3 detection channel. No proprietary software is required. Data analysis can be performed using EasyFlow or equivalent cytometry software.

## Background

The salmon aquaculture industry, primarily driven by the production of Atlantic salmon (*Salmo salar*), faces critical challenges related to early sexual maturation and the risk of escape, which can lead to significant ecological impacts through genetic introgression [1]. Triploidy induction has become established as the most commercially viable strategy to ensure the biological sterility of these populations [2]. In major producing countries such as Chile and Norway, certifying this cytogenetic status is both a regulatory and commercial requirement linked to international standards, including the Aquaculture Stewardship Council (ASC) and the Norwegian "green licenses" [3]. In Chile, guidelines issued by agencies such as SERNAPESCA emphasize the necessity of these controls to ensure the environmental sustainability of farming centers [4].

For ploidy certification, flow cytometry (FCM) remains the gold standard due to its ability to accurately quantify nuclear DNA mass [5]. However, the remote location of numerous hatcheries in isolated regions, such as Aysén or Magallanes, presents substantial logistical barriers [6]. The transport of fresh samples requires a strict cold chain (0–4°C) and minimal transit times to prevent hemolysis and DNA degradation, factors that can invalidate entire certification batches [7,8]. Given these constraints, 70% ethanol fixation at -20°C represents a practical alternative to cryopreservation, providing thermal stability and facilitating sample transport and storage under field conditions [9]. While ethanol fixation has been utilized in cytometry since the late 1980s, including early reports by Ciancio et al. (1988) [10] describing cell preservation with Hoechst 33342 and propidium iodide, its application for ploidy certification in remote aquaculture settings requires rigorous, species-specific optimization to mitigate pre-analytical variables [11].

The accuracy of FCM-based diagnostics depends fundamentally on staining stoichiometry. While simplified protocols often utilize DAPI for its convenience and DNA specificity, this ligand exhibits a significant bias toward adenine-thymine (AT)-rich regions by binding selectively to the minor groove. Biophysically, DAPI aligns with poly-AT stretches where the groove is narrow and the negative electrostatic potential is intensified [12]. Conversely, the presence of amino groups in guanine (GC regions) acts as a steric hindrance that impedes DAPI binding [13]. These variations are non-trivial; recent studies across diverse biological models have demonstrated that the choice of fluorochrome (e.g., DAPI vs. PI) can induce deviations of up to 32.9% in genome size estimation, with one-third of this error directly attributed to base bias [14]. This stoichiometric variability may reduce analytical accuracy when evaluating large and complex genomes such as those of salmonids.

In contrast, propidium iodide (PI) functions as an intercalating agent with minimal sequence preference, ensuring that fluorescence intensity is directly proportional to DNA mass. Nonetheless, the use of PI mandatorily requires the inclusion of RNase A to eliminate residual RNA in nucleated fish erythrocytes, thereby preventing false shifts in the resulting histograms. Despite these technical advantages, the deferred analysis of fixed samples introduces the risk of instrumental drift and batch effects [5,9]. Stability studies indicate that while ethanol fixation maintains optimal coefficients of variation (CV) between 3.0% and 6.5% a 4 to 72-hour window [11], equipment variability can still affect the comparability of longitudinal results [11].

In the present study, we describe an optimized protocol for *Salmo salar* fry that integrates lethal blood microsampling via caudal peduncle sectioning with a 70% ethanol fixation method (-20°C). Drawing on the preservation principles established by Ramsdorf et al. [15] and Hubálek and Flajšhans [9], we refined the staining phase using PI and RNase A to ensure stoichiometric precision. Finally, we incorporated a biological internal standardization strategy to mathematically correct for instrumental drift during deferred analysis. The application of a reference standard with a known DNA content allows for the normalization of fluorescence intensity and ensures unambiguous triploid discrimination [16].

## Method details

### Experimental desing

The study employed a randomized biological design with three independent replicates (tanks) per experimental group to ensure representativeness and minimize potential "tank effects". A total of 58 Atlantic salmon (*Salmo salar*) fry (0.126 ± 0.019 g) were initially assigned to the study, where the experimental unit was defined as the individual fish (n=1).

• Control Group (2n): n=18 individuals maintained at a preferendum temperature of 9.2 °C.
• Treatment 1 (T1, 3n): n=20 individuals subjected to a thermal shock of 28°C for 10 minutes applied 15 minutes post-fertilization to induce triploidy.
• Treatment 2 (T2, 3n): n=20 individuals subjected to a thermal shock of 28°C for 10 minutes applied 30 minutes post-fertilization to induce triploidy.

To evaluate the robustness of the flow cytometry protocol under simulated field conditions, a simple random sampling was conducted across the three biological replicates of each treatment. All samples were processed individually through lethal blood microsampling via caudal peduncle sectioning. Following the quality control phase of the flow cytometry analysis, three samples from the control group were excluded due to low cellularity, resulting in a final analytical n = 15 for diploids and n = 40 for triploids.

### Reagents and solutions

All reagents were prepared using ultrapure water (18.2 MΩ x cm).

• **Reception Buffer (Pre-bath)**: 1x Phosphate-Buffered Saline (PBS; prepared from tablets, Sigma-Aldrich, St. Louis, MO, USA, Catalog No. P4417) supplemented with 0.1% w/v Bovine Serum Albumin (BSA; Prometheus Protein Biology Products, Genesee Scientific, El Cajon, CA, USA, Catalog No. 25-529) and 5 mM Ethylenediaminetetraacetic acid (EDTA; Sigma-Aldrich, Catalog No. E5134). The solution was maintained on ice (4°C) to prevent osmotic shock and premature coagulation.
• **Fixation Solution**: Pre-cooled (-20°C) 70% absolute ethanol (ACS analysis grade, Winkler, Lampa, Chile, Catalog No. AL-0205)
• **Staining solution**: 1x PBS containing Propidium Iodide (PI, 50 µg/mL prepared from a 1 mg/mL stock solution dissolved in dimethyl sulfoxide [DMSO], Invitrogen, Thermo Fisher Scientific, Eugene, OR, USA, Catalog No. P1304MP), DNase-free RNase A (100 µg/mL; Roche Diagnostics GmbH, Mannheim, Germany, Catalog No. 10109169001), and 0.1% v/v Triton X-100 (Sigma-Aldrich). Treatment with RNase A was mandatory to eliminate residual RNA interference in nucleated erythrocytes.

### Microsampling and sample preparation

Fry were anesthetized with a benzocaine overdose (Sigma-Aldrich, Catalog No. E1501) until total loss of equilibrium. Blood microsamples (1–3 µL, varying due to the small body size of the fry) were collected via caudal peduncle sectioning using a sterile scalpel and immediate aspiration with a micropipette. Samples were deposited into 200 µL of pre-cooled “Reception Buffer” and homogenized by gentle inversion to maintain cellular integrity.

### Fixation and Storage Stability

Fixation was performed by adding pre-cooled absolute ethanol dropwise under low-speed vortexing to reach a final concentration of 70%. Samples were immediately stored at -20 °C. Flow cytometry (FCM) analysis was executed following a 4h technical stabilization period at -20 °C to allow cellular and chromatin equilibrium.

### Washing and staining procedure

Fixed samples were washed twice by adding pre-cooled PBS up to a final volume 1 mL and centrifuging at 800 x g for 5 min at 4°C. The ethanol supernatant was carefully discarded, and the pellet was resuspended in 300 µL of the “Staining Solution”. Samples were subsequently incubated for 20 minutes in the dark at room temperature (20-22°C) to allow for RNA digestion and PI intercalation. A saturating PI concentration (50 µg/mL) was utilized to ensure stoichiometric DNA binding, thereby mitigating potential fluorescence variations arising from differences in the initial blood volume (1–3 µL). Furthermore, calculating the Normalized Fluorescence Ratio (R_norm_) relative to the spiked internal standard inherently normalized any residual variance in cellular density during data acquisition.

### Internal standardization strategy

To correct for instrumental drift, laser fluctuations, and batch effects during acquisition, an internal biological standardization strategy was implemented. Prior to acquisition, 10 µL of a confirmed diploid standard was spiked into each sample tube. Crucially, this diploid internal standard must always be included in every analytical run and prepared under identical fixation, staining, and storage conditions as the test samples. This methodological pairing ensured that any pre-analytical variables or protocol-induced artifacts (e.g., alterations in chromatin compaction or variations in fluorochrome accessibility) affected both the reference and test populations equally. Consequently, this normalization strategy preserved the stoichiometric accuracy of the assay, enabling the precise calculation of the DNA Index (DI) or Normalized Fluorescence Ratio (R_norm_) as follows:

$$R_{\text{norm}}=\;\frac{\text{Mean}\;\text{Fluorescence}\;\text{Intensity}\;\left(\text{MFI}\right)\text{of}\;\text{the}\;\text{Sample}}{\text{Mean}\;\text{Fluorescence}\;\text{Intensity}\;\left(\text{MFI}\right)\;\text{of}\;\text{the}\;\text{internal}\;\text{Standard}}$$

### Flow cytometry and gating strategy

Measurements were performed using a BD Accuri C6 Plus flow cytometer (BD Biosciences, San Jose, CA, USA) equipped with a 488 nm blue laser. Red fluorescence emission was collected via the FL3 channel (>670 nm long-pass filter), with the flow rate strictly set to "Slow" (14 µL/min) to minimize the coefficient of variation (CV) and ensure optimal hydrodynamic focusing.

A minimum of 1,000 nuclear events (singlets) were acquired per sample. This acquisition threshold was analytically tailored to accommodate the low blood volume obtainable from fry (0.126 ± 0.019 g), which intrinsically restricts total cell yield. While higher event counts are common in other matrices, 1,000 events provide sufficient statistical power for reliable ploidy discrimination in teleost erythrocytes [9]. More importantly, the simultaneous acquisition of the spiked diploid internal standard permitted robust mathematical normalization of the fluorescence signal, rendering the assay insensitive to variations in absolute cell counts.

All flow cytometry standard (.fcs) data files were imported, analyzed, and visualized utilizing the Python-based open-source software EasyFlowQ (v1.7.4) [17]. This computational platform was used to execute the gating hierarchy, perform rigorous doublet exclusion, and generate high-resolution density plots (hexagonal binning) alongside single-parameter DNA histograms. The analytical gating strategy was established as follows:

1. **Primary Gating:** Forward Scatter Area (FSC-A) vs. Side Scatter Area (SSC-A) to isolate the nucleated erythrocyte population and exclude sub-cellular debris (Fig. 1A).

**Fig. 1:**
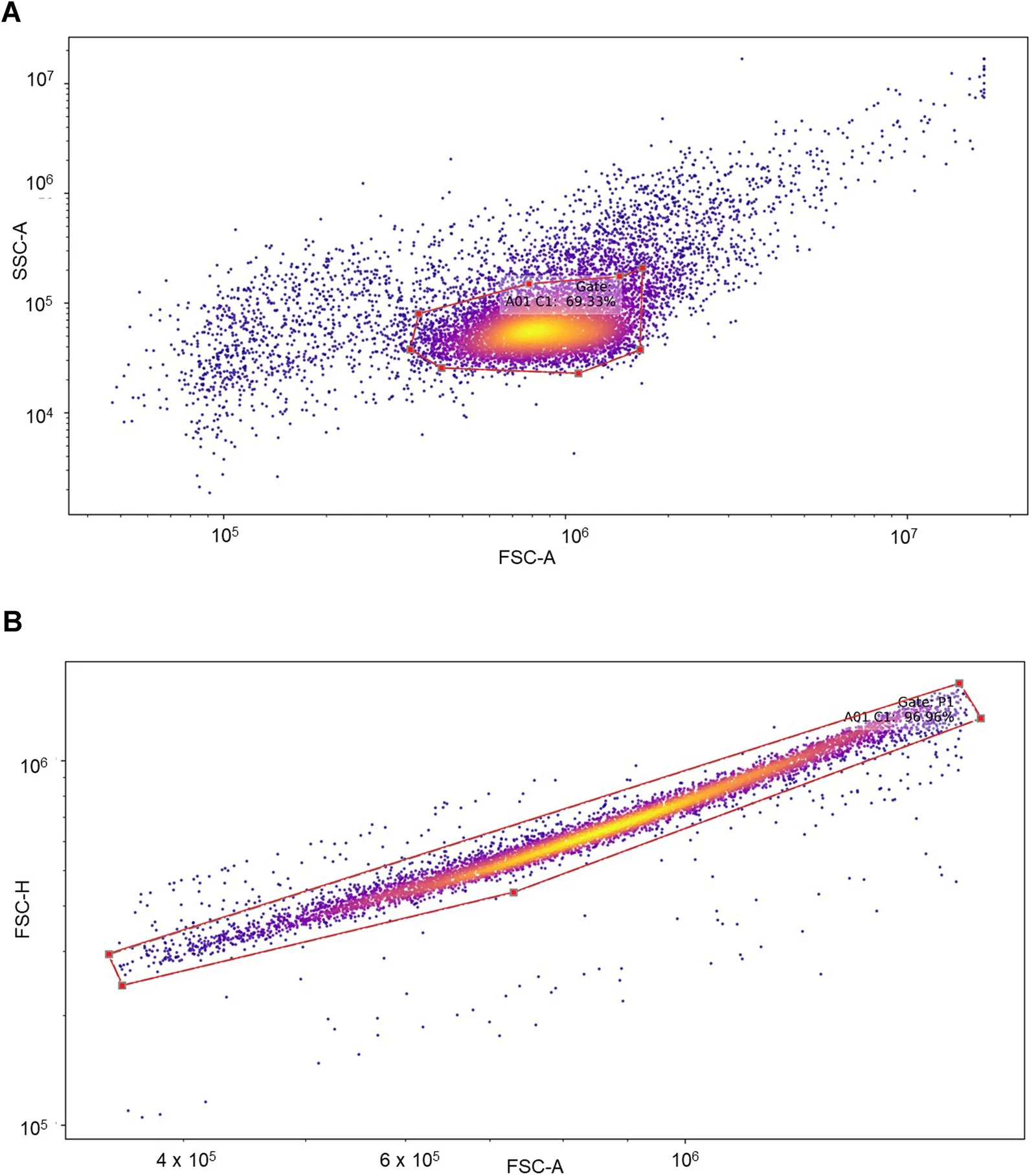
Hierarchical gating strategy for cytogenetic ploidy determination in Salmo salar blood microsamples. (A) Primary gating on a Forward Scatter Area (FSC-A) vs. Side Scatter Area (SSC-A) density plot to isolate the nucleated erythrocyte population (E3 gate, representing 69.33% of total events) and exclude subcellular debris. (B) Doublet exclusion gating on an FSC-A vs. Forward Scatter Height (FSC-H) plot of the pre-gated erythrocyte population, selecting the singlet fraction (P1 gate, representing 96.95% of cells) to prevent false polyploid signals from nuclear aggregates.
2. **Doublet Exclusion:** Forward Scatter Area (FSC-A) vs. Forward Scatter Height (FSC-H) to select single cells (singlets), preventing false polyploid peaks resulting from nuclear aggregation (Fig. 1B).

### Nuclear morphometry (validation)

Following euthanasia of the fry at 67 days post-hatching using benzocaine, a caudal fin incision was performed to obtain blood samples. Holding the specimens vertically from the head, blood was collected directly onto the edge of a glass slide to immediately prepare thin blood smears. The smears were air-dried at room temperature and subsequently fixed in pure methanol for 2 min. Cellular staining was performed using the May-Grünwald Giemsa method. The fixed smears were placed in a Coplin jar and immersed in May-Grünwald solution for 5 min. After removing the staining solution, the samples were incubated for 2 min in a 67 mM phosphate buffer (pH 6.8), prepared 6.56 g/L of KH₂PO₄ and 5.03 g/L de Na₂HPO₄ After draining the excess buffer, the slides were immersed in a 5% Giemsa solution for 10 min. Finally, the samples were rinsed with running tap water until the runoff was clear and air-dried for microscopic analysis.

Morphometric analysis was conducted using bright-field optical microscopy at 400× total magnification to delineate erythrocyte nuclear and cytoplasmic boundaries. Nuclear measurements were executed via QCapture Pro 7 software, recording the major nuclear diameter of 50 randomly selected erythrocytes per individual smear. To ensure analytical precision, erythrocytes exhibiting morphological alterations, deformation, cellular overlapping, or poorly defined nuclear margins were strictly excluded. While morphometric sampling (n = 50 nuclei per smear) differs intrinsically from high-throughput cytometric event acquisition, the appropriate unit of biological replication for this comparison is the individual fish, not the isolated nucleus. To formally support this, we fit a linear mixed model (nuclear diameter ∼ random intercept per individual) across the full nucleus-level dataset (n = 31 individuals, 1,707 measurements), yielding an intraclass correlation coefficient (ICC) of 0.62, indicating that the majority of variance in nuclear diameter arises between individuals rather than between nuclei within the same individual. At n = 50 nuclei, the intra-individual standard error of the mean was 0.086 µm, under 5% of the observed between-group difference in mean diameter, confirming that this sampling depth provides adequate precision at the organismal level. Averaging these measurements per individual yielded a robust effect size (Cohen's d = 1.11) for the diploid–triploid comparison (t = −3.13, p = 0.004).

### Statical analysis

Data distribution and homoscedasticity were assessed using the Shapiro-Wilk and Levene tests, respectively, with the latter selected for its robustness in unbalanced sample sizes (n = 15 vs. n = 40). Differences in R_norm_ and major nuclear diameter between groups were analyzed using independent samples t-tests. Statistical significance was interpreted continuously, reporting exact p-values to avoid arbitrary significance thresholds. Practical significance was supplemented by calculating Cohen's d to evaluate effect sizes.

To assess the diagnostic efficacy of nuclear morphometry compared to the flow cytometry gold standard, a Receiver Operating Characteristic (ROC) curve analysis was conducted. The Area Under the Curve (AUC) was calculated, and the optimal diagnostic threshold was determined using Youden's Index.

(J=Sensitivity+Specificity−1)

Technical quality thresholds required a measurement success rate exceeding 90% (samples with CV < 5%) and complete peak resolution between the sample and the internal standard.

## Method validation

### Measurability and precision

A total of 58 blood microsamples were processed using the 70% ethanol fixation protocol and stabilization pre-bath. The protocol achieved a measurability rate of 92.7% (n = 55/58). Three samples were excluded during the gating phase due to insufficient event counts.

**Fig. 2:**
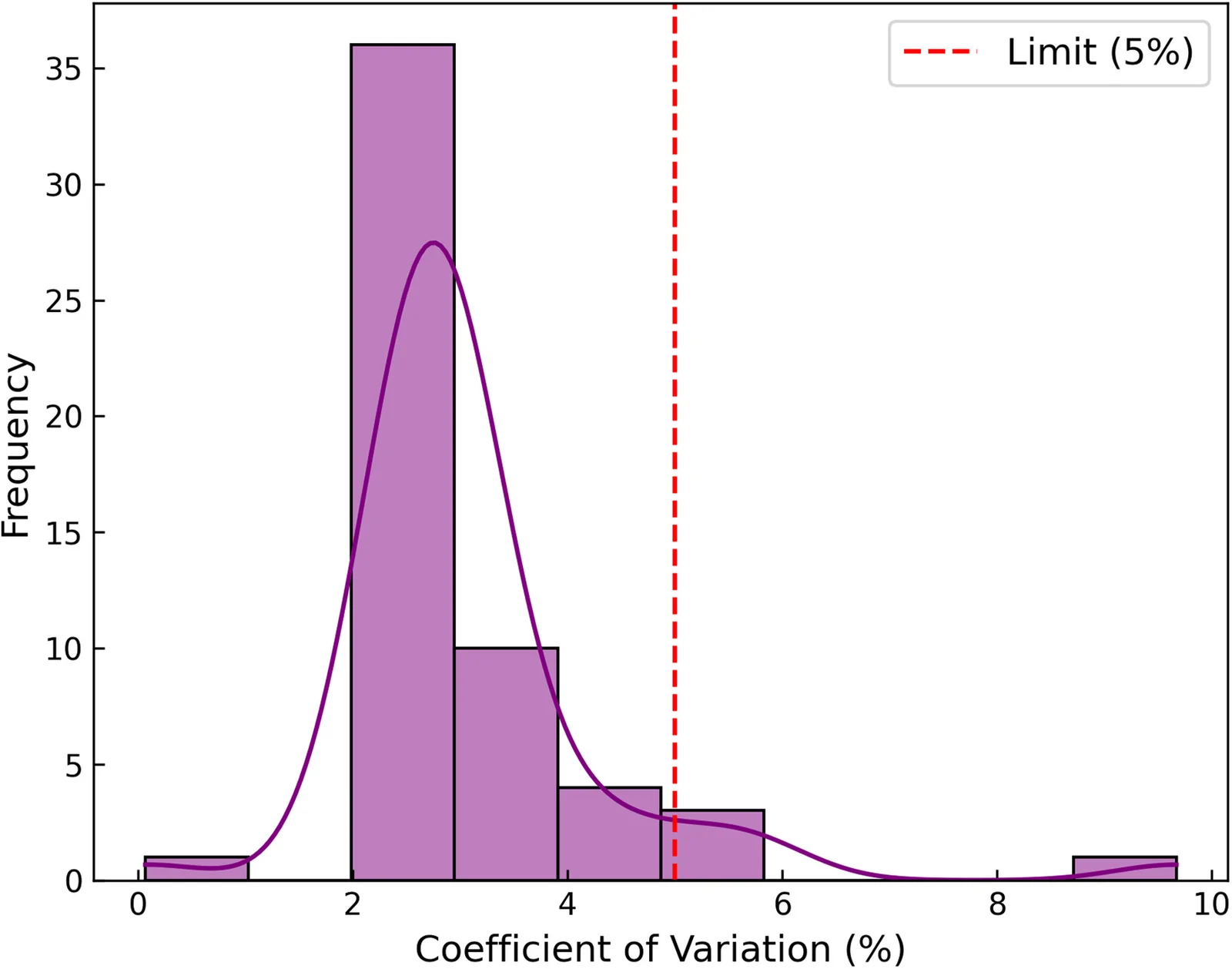
Frequency distribution of the peak Coefficient of Variation (CV, %) evaluating the technical quality and precision of the 70% ethanol fixation protocol in Salmo salar erythrocyte samples (n = 55). The red dashed line denotes the critical technical acceptance threshold (CV &lt; 5%) established by international cytometric guidelines. A total of 92.7% of the analyzed samples fall below this quality threshold, with a global mean CV of 3.10 ± 1.27%, demonstrating the robust stabilization capacity of the fixation method under simulated field conditions.s.

**Fig. 3:**
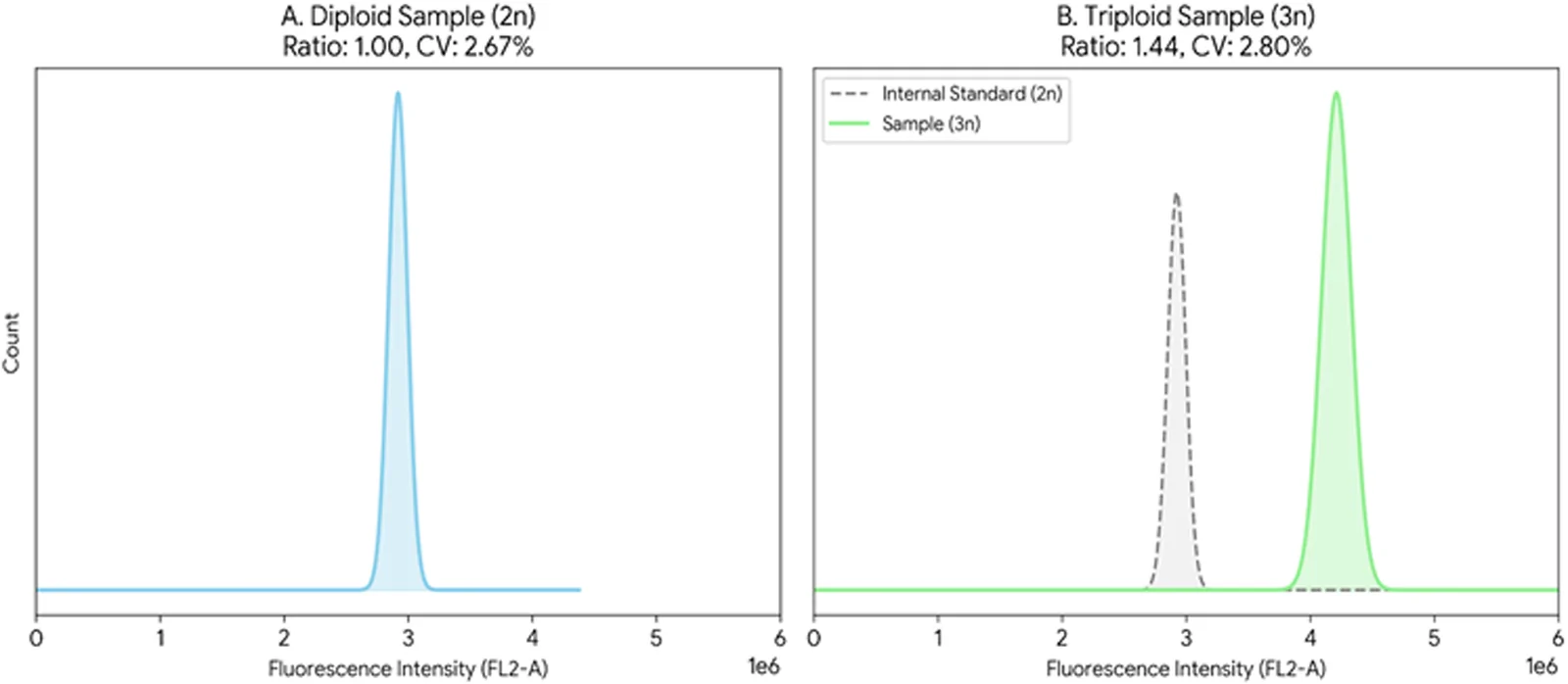
Ploidy determination workflow in Salmo salar fry using high-resolution flow cytometry. (A) Representative raw fluorescence intensity histogram (FL3-A channel, PerCP-A) of a triploid individual spiked with a diploid internal standard (sample B03 T1-3). The minor peak (3.2 × 105 A.U.) represents the diploid reference, while the major peak (4.2 × 105 A.U., M2 gate representing 77.83% of events) corresponds to the triploid population. (B) Normalized fluorescence histograms demonstrating resolution alignment for certified diploid (ratio: 1.00; CV: 2.67%) and triploid (ratio: 1.44; CV: 2.80%) individuals.

The global mean Coefficient of Variation (CV) for G0/G1 peaks was 3.10%. Regarding quality thresholds, 92.7% of the analyzed samples exhibited a CV < 5%, and 67.3% showed a CV < 3% (Fig. 2, Table 1). No significant differences were observed in CV values between diploid and triploid groups (p = 0.312). Furthermore, the high-resolution flow cytometry allowed for a clear discrimination between ploidy levels based on the normalized fluorescence ratios, as exemplified by the distinct profiles of certified diploid and triploid individuals (Fig. 3).

**Table 1:** Statistical summary of cytometric measurability and technical precision across ploidy groups in *Salmo salar*. Data represent mean values ± standard deviation (SD). The optimized chemical fixation and staining protocol achieved an overall measurement success rate of 92.7% (n = 55/58), yielding a global mean coefficient of variation (CV) of 3.10% for nucleated erythrocytes.

**Ploidy Status**	**n (Samples)**	**Mean CV ± SD (%)**	**Samples with CV < 3%**	**Samples with CV < 5%**
**Diploid**	15	3.50 ± 1.95	73.3%	86.7%
**Triploid**	40	2.95 ± 0.89	65.0%	95.0%
**TOTAL**	**55**	3.10 ± 1.27	**67.3%**	**92.7%**

Statistical summary of cytometric measurability and technical precision across ploidy groups in Salmo salar. Data represent mean values ± standard deviation (SD). The optimized chemical fixation and staining protocol achieved an overall measurement success rate of 92.7% (n = 55/58), yielding a global mean coefficient of variation (CV) of 3.10% for nucleated erythrocytes.

### Fluorescence stability and biological standardization

We detected significant variations in the absolute Mean Fluorescence Intensity (MFI) of the G0/G1 peak across different acquisition sessions (batch effect), which precluded the diagnostic utility of raw fluorescence measurements. The implementation of the spiked internal biological standard reduced the influence of instrumental fluctuations on fluorescence measurements. For the diploid control group (2n; n = 15), the Normalized Fluorescence Ratio was highly stable at 1.00 ± 0.05 (95 % confidence interval [CI]: 0.97 - 1.03), demonstrating negligible variation in relative fluorescence in ethanol-fixed cells relative to the internal standard.

For the overall triploid cohort (3n; n = 40), the ratio stabilized at 1.38 ± 0.04 (95 % CI: 1.36 -1.40). This relative shift from the theoretical 1.50 value is consistent with chromatin compaction variations in polyploid cells. When evaluating the specific polyploidization windows, the T1 sub-group (thermal shock applied 15 min post-fertilization; n = 20) exhibited an R_norm_ of 1.39 ± 0.03, while the T2 sub-group (thermal shock applied 30 min post-fertilization; n = 20) showed an R_norm_ of 1.37 ± 0.04. No statistically significant differences in R_norm_ or CV values were detected between the two induction times (p > 0.05).

**Fig. 4:**
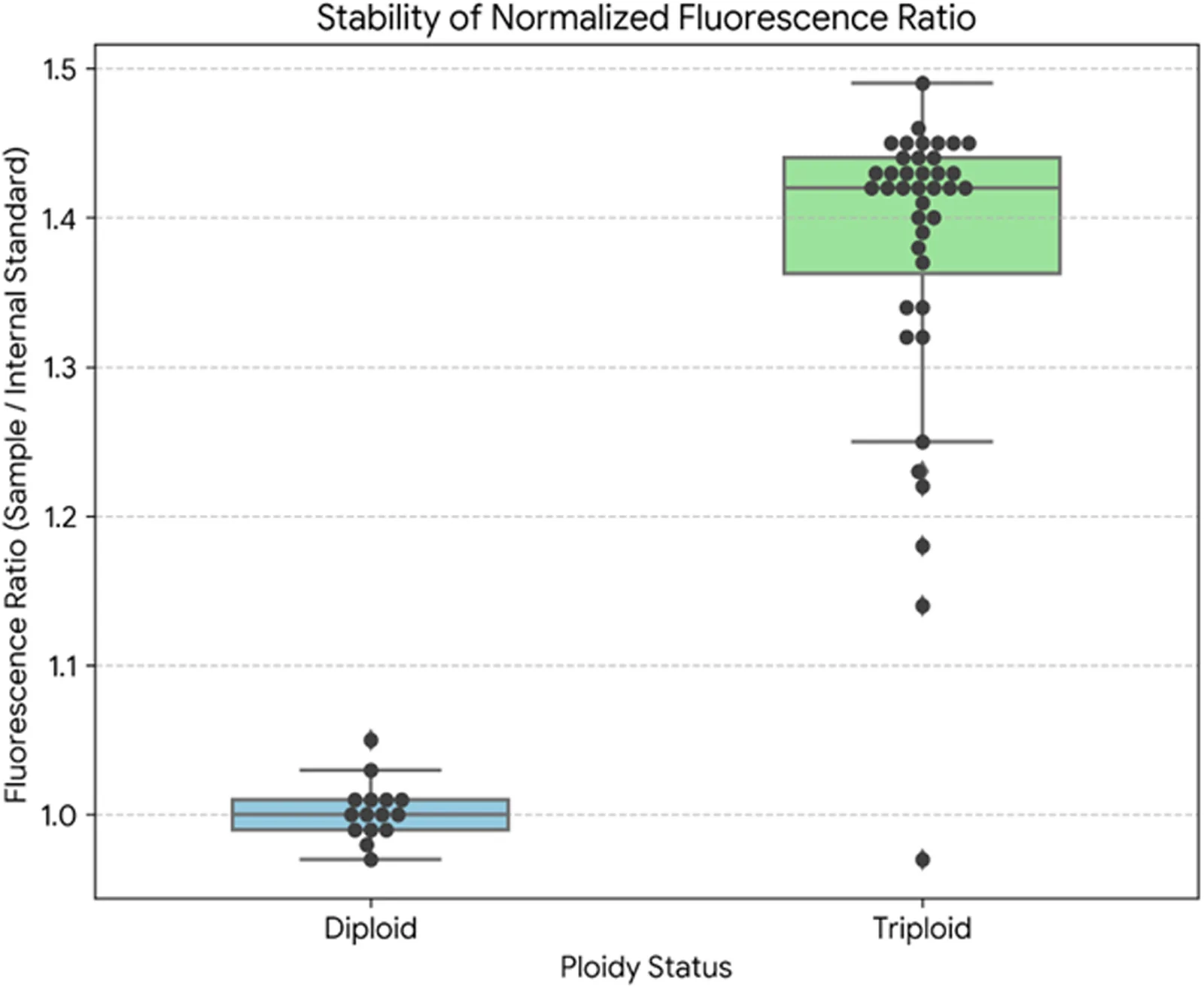
Distribution of the Normalized Fluorescence Ratio (Rnorm) for ploidy determination in Salmo salar erythrocytes. Boxplots with overlaid individual data points show complete segregation between diploid (n = 15) and triploid (n = 40) cohorts. The Rnorm was stable at 1.00 ± 0.05 for diploids and 1.38 ± 0.04 for triploids (mean ± SD). The deviation of triploids from the theoretical 1.50 ratio is consistent with chromatin compaction in polyploid cells. Horizontal lines represent medians, box limits indicate the interquartile range (IQR), and whiskers extend to 1.5 x IQR.

To facilitate practical interpretation of the results, operational cut-off thresholds were established based on three standard deviations (3SD) from the observed means. The operational acceptance range for certified diploid status was defined between 0.85 and 1.15, whereas the acceptance range for certified triploid status was established between 1.26 and 1.50. The R_norm_ distributions of the 2n control and the 3n induction groups (including both T1 and T2 sub-groups) demonstrated clear separation with no observed overlap (Fig. 4). This distinct segregation yielded a diagnostic sensitivity, specificity, and positive/negative predictive values of 100% across the analyzed samples.

### Nuclear morphometric validation

**Fig. 5:**
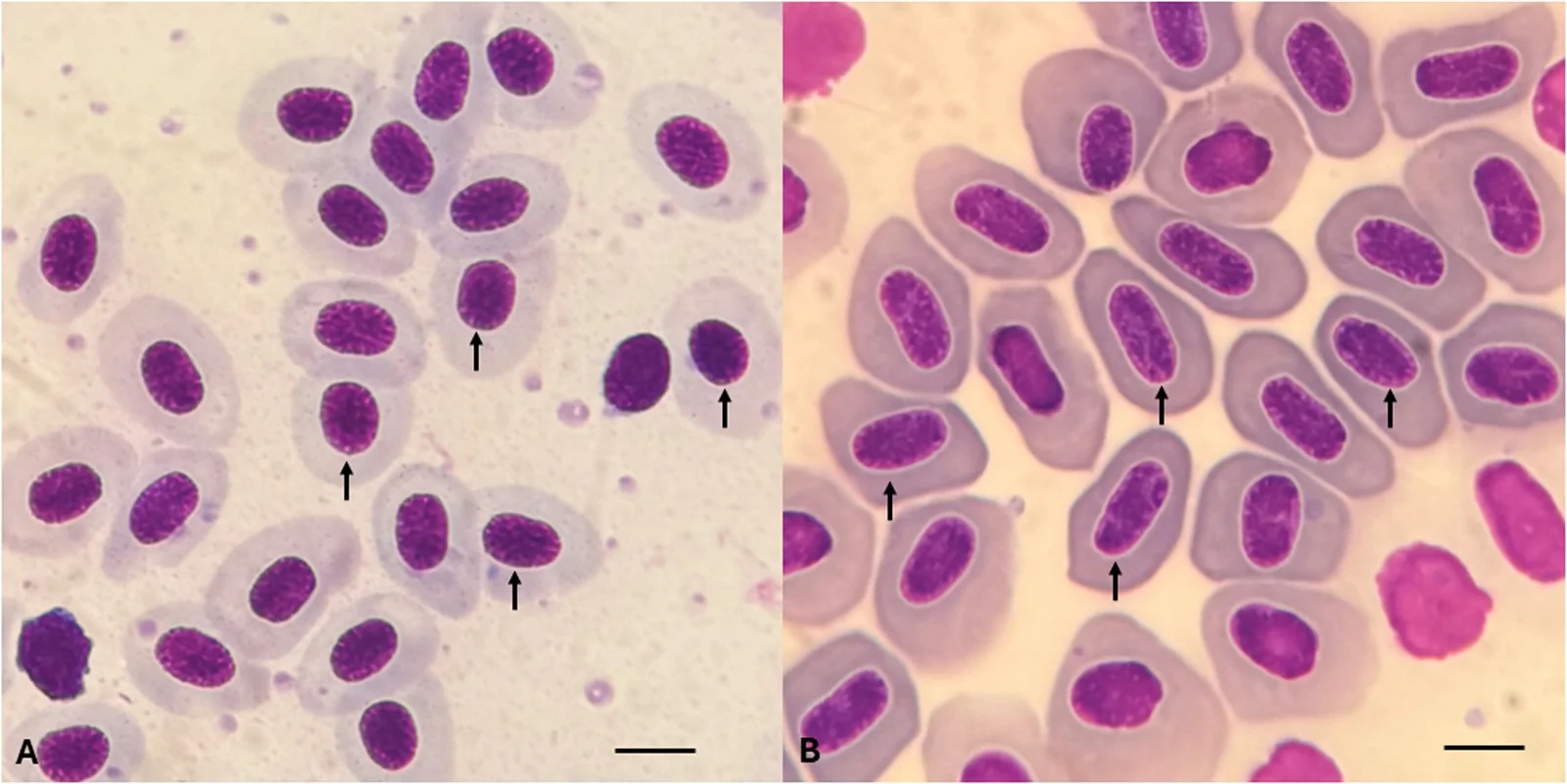
Nucleated erythrocytes (arrows) from circulating blood in Atlantic salmon fry (Salmo salar). A. Diploid control. B: Triploid treatment. Total magnification: 1000×.

**Fig. 6:**
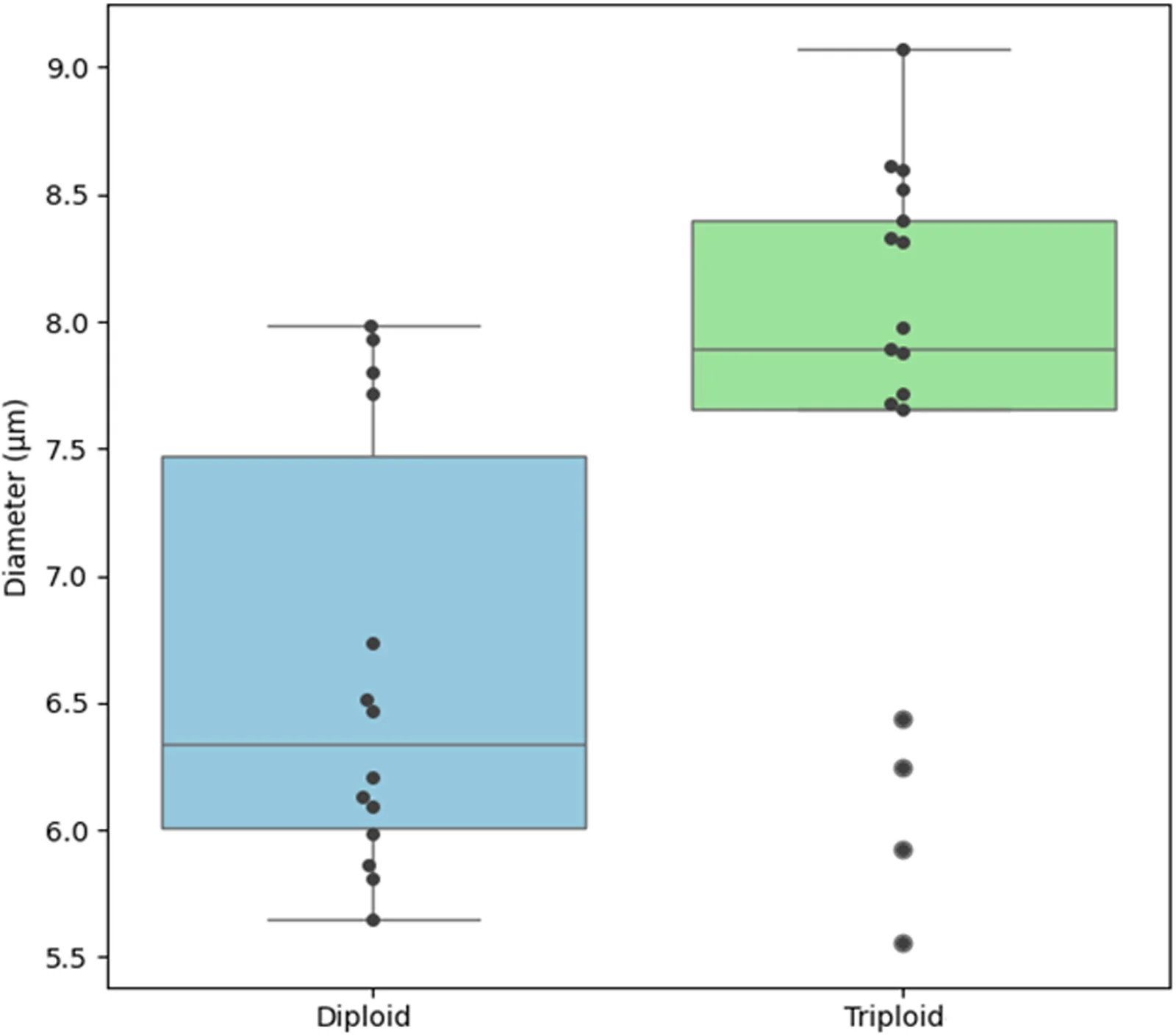
Comparative distribution of the major nuclear diameter (µm) between diploid (2n, n = 14) and triploid (3n, n = 17) individuals. Although a statistically significant enlargement is present in triploids (7.69 ± 1.04 µm vs. 6.63 ± 0.83 µm; p = 0.004; Cohen’s d = 1.12), the marked distribution overlap between 7.0 and 8.0 µm limits the diagnostic accuracy of this method.

Image analysis of fixed Giemsa-stained blood smears (n = 31) confirmed a distinct nucleotypic effect associated with chromosome set duplication (Fig. 5). Diploid erythrocytes exhibited a mean major nuclear diameter of 6.63 ± 0.83 µm, while triploids displayed a significantly larger nuclear diameter of 7.69 ± 1.04 µm (Fig. 6).

Statistical evaluation confirmed a significant size increase (independent samples t-test, exact p = 0.004) with a large effect size (Cohen’s d = 1.12). However, a prominent size overlap was observed between the two populations, particularly within the 7.0 - 8.0 µm range.

**Fig. 7:**
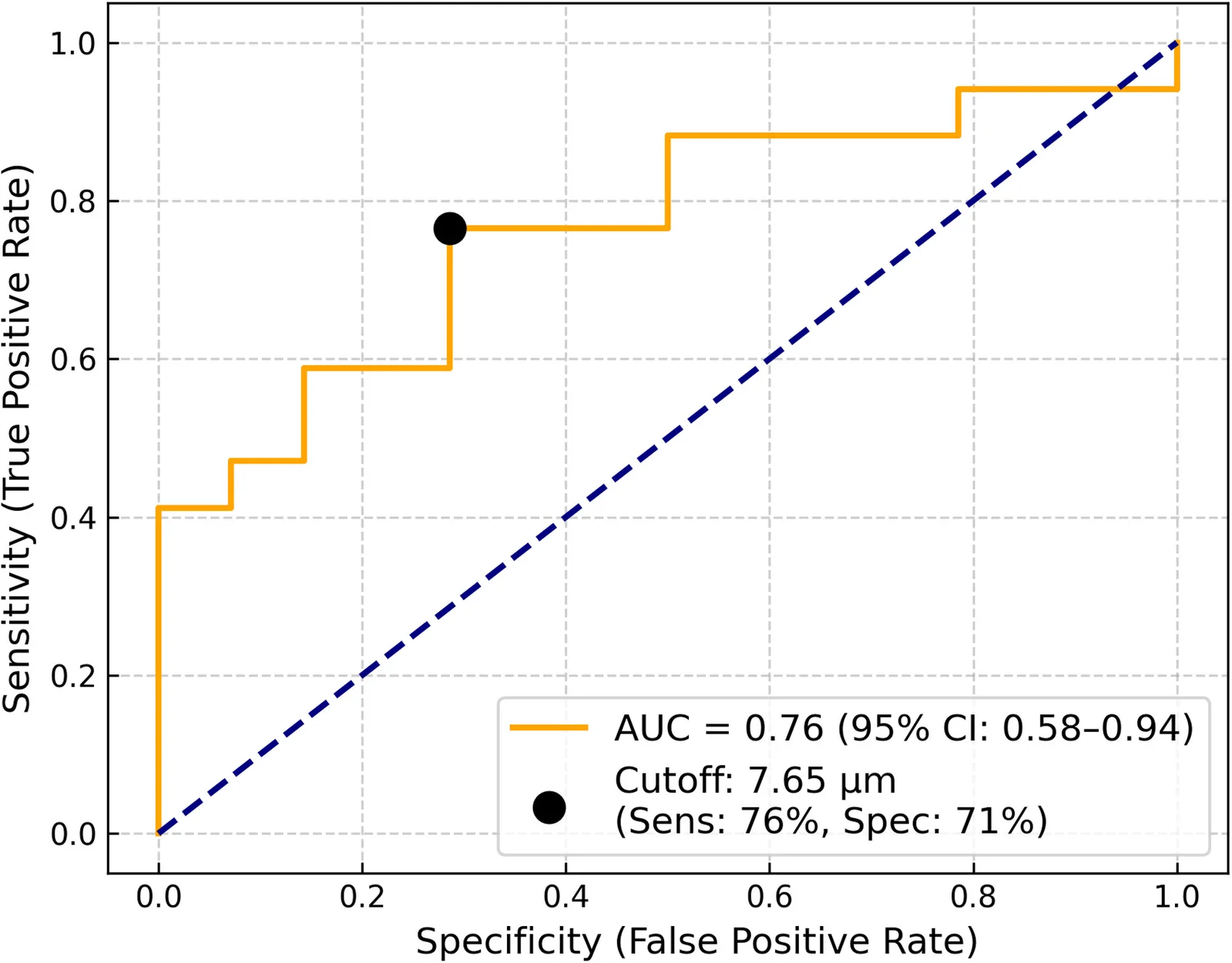
Receiver Operating Characteristic (ROC) curve evaluating the diagnostic accuracy of nuclear morphometry for ploidy determination, using flow cytometry as the reference standard. The Area Under the Curve (AUC) was 0.76. The optimal diagnostic threshold was determined to be 7.65 µm using Youden’s Index, yielding a sensitivity of 76% and a specificity of 71%, which corresponds to a diagnostic uncertainty zone of 24%.

To evaluate the diagnostic reliability of nuclear morphometry against the flow cytometry gold standard, a Receiver Operating Characteristic (ROC) curve analysis was conducted. The Area Under the Curve (AUC) was 0.76 (95% confidence interval: 0.58–0.94). Using Youden’s Index, the optimal diagnostic cut-off was established at 7.65 µm which achieved a sensitivity of 76% and a specificity of 71%. Consequently, while nuclear morphometry serves as an accessible screening tool, it exhibits a 24% diagnostic uncertainty zone, highlighting the necessity of flow cytometry for official ploidy certification (Fig. 7).

### Performance of ethanol fixation for ploidy analysis

The global mean CV of 3.10 ± 1.27 % obtained in this study aligns with the resolution standards typically discussed for ploidy discrimination in salmonids, where a threshold of <5% is generally recommended for reliable certification. While earlier research by Yasui et al. [18], emphasized the importance of ultra-low or cryogenic temperatures to prevent DNA fragmentation in dish samples, the results obtained in the present study indicate that 70% ethanol fixation at -20°C preserved sample quality sufficiently for ploidy determination.

Furthermore, the analytical success rate (92.7%) achieved following a 4-hour technical stabilization period exhibited a diagnostic performance comparable to standard protocols utilizing fresh tissue. This efficacy is fundamentally associated with the biochemical nature of ethanol as a coagulant fixative; unlike aldehyde-based alternatives, ethanol prevents covalent protein-DNA cross-linking, thereby ensuring that fluorochrome intercalation sites remain sterically accessible [6].

While the extended storage viability (24–72 h) of ethanol-fixed teleost blood for field transport has been previously validated by Hubálek and Flajšhans [9], the 4-hour stabilization period in the present study was specifically designed to establish the baseline performance of our optimized PI/RNase A staining solution and internal standardization strategy in *Salmo salar*. Although the established fixative principles robustly support long-term transport logistics, future longitudinal studies are warranted to confirm the stability of this mathematically corrected PI-fluorescence ratio over extended field-storage intervals.

### Stoichiometric superiority: PI intercalation vs. DAPI Bias

The methodological transition from DAPI to Propidium Iodide (PI) addresses a well-documented biophysical constraint in salmonid cytogenetics. DAPI binds to the minor groove of DNA with a strict preference for adenine-thymine (AT) rich regions [12,13]. Although significant deviations in DNA content estimation have been characterized in complex genomes due to this AT-bias [14], the principle remains relevant when analyzing the pseudotetraploid genome of *S. salar.*

The use of an intercalating agent like PI minimizes sequence-selective bias; as noted by Zhu et al. in fish breeding applications, non-selective intercalators can align fluorescence intensity more closely with total DNA mass, regardless of regional base-pair variations [11]. This characteristic, combined with the inclusion of RNase A to reduce residual RNA signal in nucleated erythrocytes, contributes to mitigating histogram noise and potential shifts in the DNA index without relying on preferential binding mechanisms [5].

### Internal Standardization and the 1.38 ratio variance

The observed fluctuations in raw Mean Fluorescence Intensity (MFI) between sessions confirm that instrumental drift remains a significant barrier to routine ploidy diagnostics. Our implementation of a spiked internal biological standard successfully mitigated this drift, serving as an internal reference for data normalization. The resulting Normalized Fluorescence Ratio (NFR) of 1.38 ± 0.04 for triploids (while lower than the theoretical 1.5) is consistent with patterns observed in ethanol-fixed fish cells. This deviation may be associated with differential chromatin compaction and nuclear dehydration in polyploid cells, which could reflect differences in chromatin organization and slightly reduce fluorochrome accessibility compared to diploid controls [9,16]. However, this mechanistic hypothesis requires future validation through ultrastructural studies at the chromatin level. Nevertheless, the distinct separation observed between the NFR distributions of 2n and 3n populations allowed for reliable ploidy discrimination across the analyzed samples [11].

### Morphometric limitations and uncertainty zones

Cross-validation via nuclear morphometry confirmed a significant increase in nuclear diameter (7.69 ± 1.04 µm), reflecting the cellular volume expansion derived from the retention of the second polar body [2]. However, the achieved AUC of 0.76 demonstrates a moderate diagnostic capacity rather than a high-resolution performance. The prominent dimensional overlap between the 2n and 3n nuclear sizes, particularly within the 7.0 - 8.0 µm range, establishes a 24% limited diagnostic resolution, restricting the sensitivity to 76% and the specificity to 71%. These parameters indicate that while nuclear morphometry serves as an accessible screening tool, its analytical limitations prevent it from being used as an independent method for official ploidy certification [19].

Regarding cytometric performance, while the inherent logistical constraints of remote sampling precluded the inclusion of a parallel fresh-blood control, the efficacy of the optimized protocol is strongly supported by literature benchmarking. The global mean CV of 3.10% achieved via ethanol fixation falls strictly within the optimal resolution standards reported for fresh salmonid erythrocytes (typically 2.5–5.0%) [9,19]. This demonstrates that targeted ethanol preservation, when coupled with mathematical standardization, does not compromise analytical resolution. Given that maintaining a strict 0–4°C cold chain is logistically prohibitive for decentralized hatcheries in geographically isolated regions (such as Aysén and Magallanes) this optimized fixation strategy represents a highly robust, and often the only practical, alternative to ensure regulatory compliance.

### Industrial implications and genetic containment

Optimizing this protocol for fry offers an alternative approach for early-stage ploidy monitoring, which is a relevant component in contemporary aquaculture management [4]. Compared to methodologies relying on strict cold chains (0–4°C) for fresh transport, the proposed procedure provides a decentralized sampling option that can simplify field logistics in geographically isolated areas.

In a regulatory landscape where minimizing diploid presence is critical for international standards such as the ASC or "green licenses", achieving a clear diagnostic segregation and a low CV represents a useful tool for production monitoring [3]. By facilitating stable transit from remote regions, this workflow contributes to the technical tools available for verifying the biological sterility of farmed populations, aiding in the management risks associated with potential genetic introgression into wild populations [1,6].

From an economic and operational perspective, fry-based ploidy assessment requires significant resource allocation (including feeding, rearing, and infrastructural footprint) prior to lethal sampling. An alternative strategy involves ploidy determination at the embryonic stage (ideally within the first week post-fertilization), which enables the early identification and culling of sub-optimal triploid batches before grow-out begins. Recent advances, such as the rapid, enzyme-free protocol for isolating embryonic cells described by Golotin et al. [20], offer promising avenues compatible with both field conditions and flow cytometry. Such approaches successfully circumvent the logistical incompatibilities of conventional enzyme-dependent methods, such as the pronase-based dechorionation protocols extensively utilized in zebrafish (*Danio rerio*) models [21]. However, extrapolating these embryonic techniques to *Salmo salar* necessitates rigorous empirical validation to confirm their analytical reliability and accuracy in salmonid matrices. Until such emerging tools are fully optimized to guarantee the diagnostic stringency required for salmonid certification, the fry-based protocol validated herein remains indispensable for commercial operations. Consequently, future research should quantitatively analyze the diagnostic fidelity and cost-efficiency of these novel embryonic screening tools relative to established fry-based frameworks under industrial-scale conditions.

## Limitations

This study indicates that the integrated combination of 70% ethanol fixation at −20°C, Propidium Iodide (PI) and RNase A staining, and an internal biological standardization strategy evaluated via the Normalized Fluorescence Ratio (R_norm) represents an alternative approach for *Salmo salar* ploidy monitoring. This combined approach enabled discrimination between diploid (2n) and triploid (3n) cohorts, mitigating DAPI stoichiometric constraints and controlling for instrumental fluctuations. While the evaluated 4-hour technical stabilization period yielded high-quality resolution with a mean Coefficient of Variation of 3.10 ± 1.27%, meeting standard laboratory precision requirements, the operational applicability of this workflow under extended transport conditions (24–72 h) requires further validation, and future studies should evaluate PI-fluorescence ratio stability over longer storage windows.

Additionally, cross-validation via nuclear morphometry confirmed the expected nucleotypic enlargement in triploids (7.69 ± 1.04 µm), though its diagnostic uncertainty zone of 24% underscores the limitations of the morphometric approach as an independent tool for regulatory certification. While field-imposed logistical constraints precluded parallel fresh-blood controls, and fry microsampling intrinsically restricted the acquisition yields to 1,000 cytometric events and 50 morphometric nuclei per individual, these constraints were addressed through complementary lines of evidence rather than a single metric. The mathematical normalization provided by the spiked internal standard mitigated residual cellular density variance, and at the morphometric level, a linear mixed-model analysis (ICC = 0.62) confirmed that the majority of variance in nuclear diameter arose between individuals rather than between nuclei within the same fish, with an intra-individual standard error of 0.086 µm at n = 50 nuclei, supporting the adequacy of these sample sizes for this matrix, while the modest total number of individuals remains a constraint on diagnostic precision that larger cohorts in future work would help address.

Future investigations must quantitatively analyze the stability of the PI-fluorescence ratio over extended field-transport storage windows (24–72 h). Prospective research should also prioritize multi-center operational validation, expanding the methodology to other commercially relevant salmonid species, automating the computational analysis pipeline, and evaluating its definitive integration into international regulatory certification frameworks.

## Ethics statements

This study followed all guidelines described in the Guide for the Care and Use of Laboratory Animals (National Research Council, 2011) and was reviewed and approved by the Animal Ethics Committee of the Catholic University of Temuco (approval number N° CEIUCT 0420001/ 23).

## CRediT authorship contribution statement

**Sebastián Ávila:** Conceptualization, Methodology, Validation, Formal analysis, Investigation, Data curation, Writing – original draft, Writing – review & editing, Visualization, Supervision. **Maira Toro:** Investigation, Methodology, Validation. **Wellison Amorim Pereira:** Methodology, Formal analysis, Writing – review & editing. **Christian Ellwanger-Reyes:** Methodology, Validation, Writing – review & editing. **Iván Valdebenito Isler:** Methodology, Validation, Writing – review & editing. **Elías Figueroa-Villalobos:** Conceptualization, Methodology, Validation, Resources, Data curation, Writing – review & editing, Supervision, Project administration, Funding acquisition.

## Declaration of competing interest

The authors declare that they have no known competing financial interests or personal relationships that could have appeared to influence the work reported in this paper.

## Data Availability

Data will be made available on request.
